# Glaucoma and intraocular pressure in EPIC-Norfolk Eye Study: cross sectional study

**DOI:** 10.1136/bmj.j3889

**Published:** 2017-09-13

**Authors:** Michelle P Y Chan, David C Broadway, Anthony P Khawaja, Jennifer L Y Yip, David F Garway-Heath, Jennifer M Burr, Robert Luben, Shabina Hayat, Nichola Dalzell, Kay-Tee Khaw, Paul J Foster

**Affiliations:** 1 Division of Genetics and Epidemiology, UCL Institute of Ophthalmology, London EC1V 9EL, UK; 2 Department of Ophthalmology, Norfolk and Norwich University Hospital NHS Foundation Trust, Norwich NR4 7UY, UK; 3 University of East Anglia, Norwich NR4 7TJ, UK; 4 Department of Public Health and Primary Care, University of Cambridge, Cambridge CB1 8RN, UK; 5 NIHR Biomedical Research Centre Moorfields Eye Hospital NHS Foundation Trust, London EC1V 2PD, UK; 6 UCL Institute of Ophthalmology, London EC1V 9EL, UK; 7 School of Medicine, Medical and Biological Sciences, University of St Andrews, St Andrews KY16 9TF, Scotland, UK

## Abstract

**Objectives** To report the distribution of intraocular pressure (IOP) by age and sex and the prevalence of glaucoma.

**Design** Community based cross sectional observational study.

**Setting** EPIC-Norfolk cohort in Norwich and the surrounding rural and urban areas.

**Participants** 8623 participants aged 48-92 recruited from the community who underwent ocular examination to identify glaucoma.

**Main outcome measures** Prevalence and characteristics of glaucoma, distribution of IOP, and the sensitivity and specificity of IOP for case finding for glaucoma.

**Results** The mean IOP in 8401 participants was 16.3 mm Hg (95% confidence interval 16.2 mm Hg to 16.3 mm Hg; SD 3.6 mm Hg). In 363 participants (4%), glaucoma was present in either eye; 314 (87%) had primary open angle glaucoma. In the remaining participants, glaucoma was suspected in 607 (7%), and 863 (10.0%) had ocular hypertension. Two thirds (242) of those with glaucoma had previously already received the diagnosis. In 76% of patients with newly diagnosed primary open angle glaucoma (83/107), the mean IOP was under the threshold for ocular hypertension (21 mm Hg). No one IOP threshold provided adequately high sensitivity and specificity for diagnosis of glaucoma.

**Conclusions** In this British community, cases of glaucoma, suspected glaucoma, and ocular hypertension represent a large number of potential referrals to the hospital eye service. The use of IOP for detection of those with glaucoma is inaccurate and probably not viable.

## Introduction

Glaucoma is the leading cause of irreversible blindness in the world^1^ and the second most common cause of registered blindness in England and Wales.^2^ It comprises a group of ocular diseases of progressive damage to the optic nerve, with characteristic structural changes to the optic disc and visual field defects.^3^ Glaucoma and suspected glaucoma combined account for the sixth largest share of National Health Service (NHS) outpatient attendances in England, after general medical examination, breast cancer, schizophrenia, prostate cancer, and joint pain.^4^ The most common type of glaucoma among white people is primary open angle glaucoma (POAG); primary angle closure glaucoma (PACG), which results from occlusion of aqueous humour outflow, is more common among Asian people.^5^ Secondary glaucoma results from a diverse range of ocular and systemic conditions. Raised intraocular pressure (IOP) is the major modifiable risk factor for primary open angle glaucoma,^6^ ^7^ ^8^ but around half of people with glaucoma present with IOP below 21 mm Hg, which is the threshold for ocular hypertension (raised IOP without any evidence of glaucoma).^9^ The EPIC-Norfolk Eye Study, initiated in 2004, is the most recent large scale eye survey in the UK. We examined the prevalence and characteristics of glaucoma and distribution of IOP in the study participants.

## Methods

The European Prospective Investigation of Cancer (EPIC) study is a pan-European multi-cohort study designed to investigate the lifestyle determinants of risk of cancer. The EPIC-Norfolk cohort was established in the city of Norwich and the surrounding rural and urban areas, in the eastern English county of Norfolk, in 1993-97.^10^ A total of 30 445 men and women aged 40-79 were recruited at a baseline survey from the databases of 35 general practices. The predominant ethnicity of the cohort was white, and it included individuals across the range of socioeconomic status and educational achievements. The EPIC-Norfolk Eye study was carried out in 2004-11, when ophthalmic data were collected from 8623 participants.^11^

The first 443 sequential participants had IOP measured with a non-contact tonometer (AT555, Reichert Corporation, Philadelphia, PA, USA). In the remaining participants IOP was measured three times in each eye with the ocular response analyser (ORA) non-contact analyser (Reichert Corporation, Philadelphia, PA, USA) with software version 3.01. This flattens the cornea with a jet of air and uses an electro-optical system to measure the air pressures at which the cornea flattens both inwards and outwards. The average of the two pressure values are calibrated linearly against the Goldmann applanation tonometer (GAT) to provide a Goldmann-equivalent IOP measurement (IOPg, mm Hg).^12^

A systematic review showed that among 12 studies that directly compared the agreement between IOPg and GAT, the mean difference between the two (IOPg−GAT) is 1.5 mm Hg (95% predicted interval −0.6 mm Hg to 3.7 mm Hg).^13^

The glaucoma status of the participants was determined from a systematic examination that included visual acuity, tonometry, and assessment of the optic nerve head (Heidelberg Retina Tomograph II) and the peripapillary nerve fibre layer with scanning laser polarimetry (GDx VCC, Zeiss, Dublin, CA, USA). A 24-2 central threshold visual field test (Humphrey 750i Visual Field Analyzer, Carl Zeiss Meditech, Welwyn Garden City, UK) was performed in those participants with abnormal findings on HRT or GDx VCC and in one in 10 with normal findings. Those with abnormal findings who met a set of predefined criteria designed to detect glaucoma were referred to the eye department of the Norfolk and Norwich University Hospital for a definitive eye examination by a consultant ophthalmologist with a specialist interest in glaucoma (DCB). A detailed description of the study design has been published previously.^11^ Glaucoma was defined as the presence of characteristic structural abnormalities of the optic disc and visual field loss, with no other explanations for the disc and field appearances. The differentiation between high tension and normal tension glaucoma was based on IOP level before glaucoma treatment started. Suspected glaucoma was defined as the presence of early or minor glaucomatous disc features, associated with a normal visual field or the absence of visual field data. Ocular hypertension was defined as IOP >21 mm Hg with no features of glaucoma in the optic disc or visual field. Specific quantitative methods and principles for diagnosis of primary open angle glaucoma and suspected primary open angle glaucoma followed the diagnostic principles from the International Society of Geographical and Epidemiological Ophthalmology (ISGEO).^3^ To limit false positive or false negative results, another consultant glaucoma ophthalmologist (PJF) reviewed all examination findings and history in a subset of high risk participants. Figure 1 shows the flow of participants through the study and the diagnostic process. We determined glaucoma diagnosis per person by taking the clinically more serious diagnosis of either eye in the following hierarchy (most serious to least serious): glaucoma, suspected glaucoma, ocular hypertension (IOP >21 mm Hg), narrow angle spectrum (primary angle closure, primary angle closure suspect and narrow angles), and normal.

**Figure f1:**
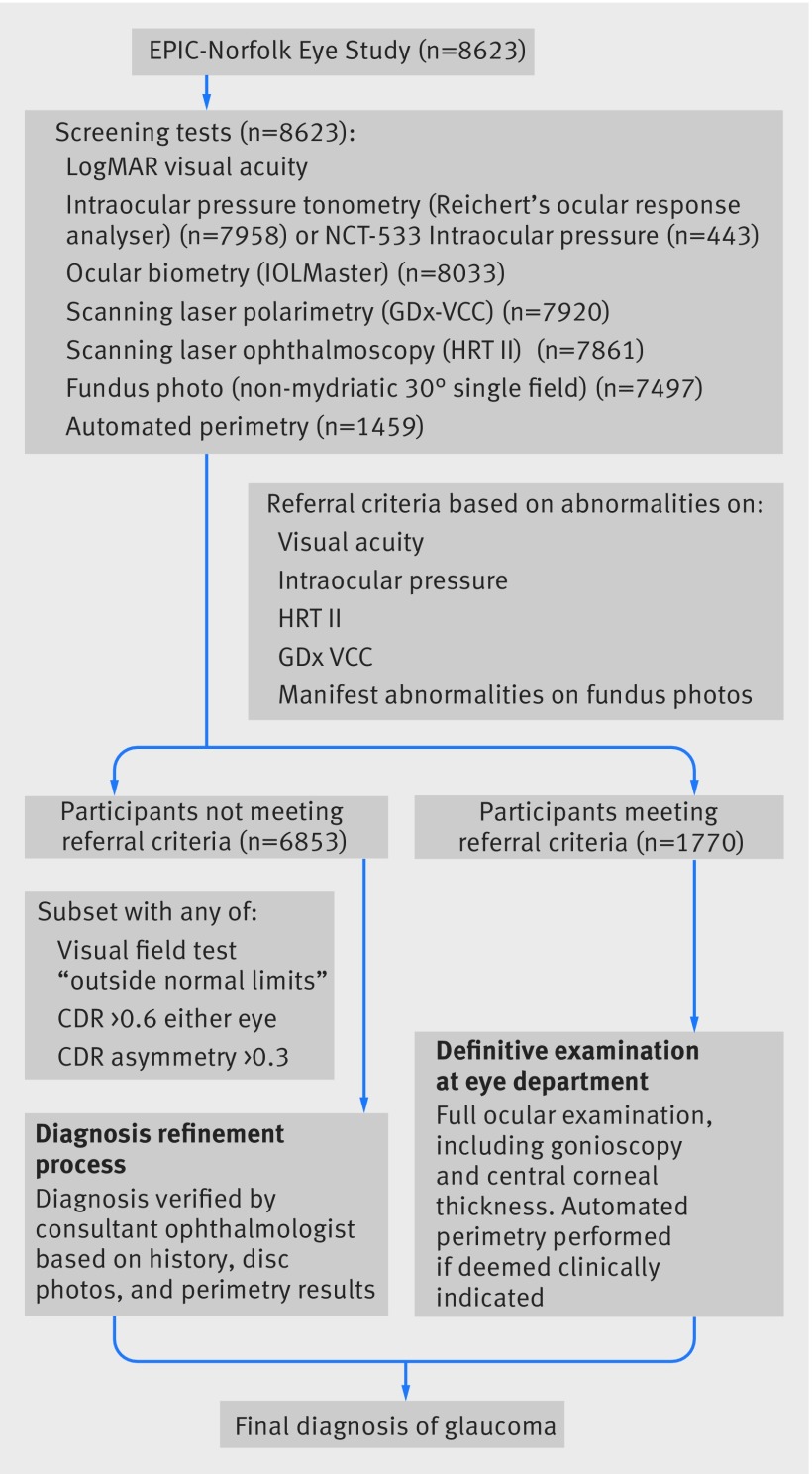
**Fig 1** Flow of participants through EPIC-Norfolk study

### Statistical analysis

The IOP reported for the cohort was the mean of the mean IOP in the left and right eyes, with the ORA IOPg or the AT555 NCT values. We calculated sensitivities and specificities of IOP for glaucoma detection from the ability of various IOP thresholds to differentiate between participants with all cause glaucoma in either eye and those with no glaucoma in either eye. The reporting of this study conformed to the STROBE statement.^14^ All statistical analyses were performed with STATA (Stata/SE 13.1, StataCorp, College Station, TX).

## Results

There were 8623 participants in the EPIC-Norfolk Eye Study, with a mean age of 68.7 (range 48-92), and over half (55%) were women. Compared with the population estimates for Norfolk and for the UK, the study population was older and had a decreasing proportion of women with age, which is opposite to the Norfolk and UK population’s trend of an increasing proportion of women with age (fig 2). Nearly all participants were white (99.4%), compared with 96.5% and 87.2%, respectively, in Norfolk and the UK.^15^

**Figure f2:**
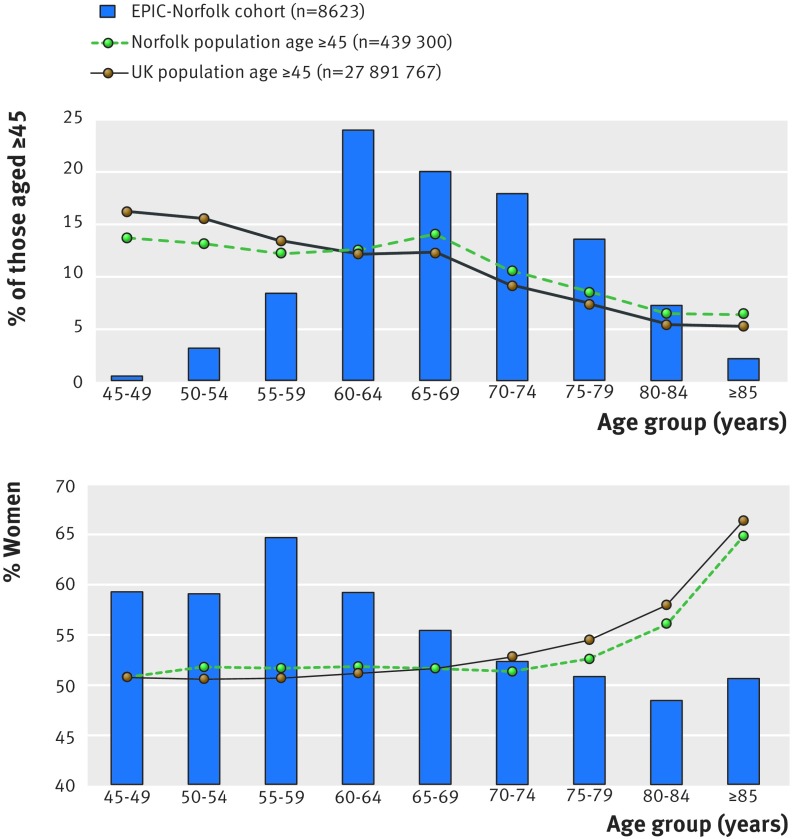
**Fig 2** Age and sex distribution of EPIC-Norfolk 3HC cohort compared with population of Norfolk and UK (2014 mid-year population estimates^15^)

Tables 1 and 2[Table tbl1 tbl2] show the glaucoma diagnosis by eye and by person. A total of 363 participants (4.2%, 95% confidence interval 3.8% to 4.6%) had glaucoma in either eye, 314 had primary open angle glaucoma (3.6%, 3.3% to 4.0%), 607 (7.0%) had suspected glaucoma, 863 (10.0%) had ocular hypertension (untreated IOP >21 mm Hg), and 54 (0.6%) had narrow angle spectrum. Twenty three participants (0.3%) had no recorded diagnosis as they declined or were unable to undergo definitive eye examination after abnormal results on the screening tests. Table 3 breaks down glaucoma by type in the 363 affected men and women. Most people with glaucoma had primary open angle glaucoma (86.5%), with an equal proportion of high pressure and normal pressure glaucoma. Out of the 523 eyes affected by glaucoma, formal visual field assessment was not feasible in 28 because of poor vision. Most of these participants had secondary glaucoma, which was diagnosed by advanced disc cupping and uncontrolled IOP.

**Table 1 tbl1:** Diagnosis of glaucoma by eye in 8623 men and women aged 48-92 in EPIC-Norfolk cohort. Figures are numbers (percentage) of participants

Diagnosis	Right eye	Left eye
Normal	7091 (82.2)	7061 (81.9)
Primary open angle glaucoma	236 (2.7)	230 (2.7)
High tension glaucoma	121 (1.4)	121 (1.4)
Normal tension glaucoma	115 (1.3)	109 (1.3)
Primary angle closure glaucoma	20 (0.2)	17 (0.2)
Secondary glaucoma	9 (0.1)	11 (0.1)
Subtotal with glaucoma	265 (3.1)	258 (3.0)
Suspected open angle glaucoma	444 (5.2)	443 (5.1)
Ocular hypertension and suspected open angle glaucoma	67 (0.8)	67 (0.8)
Suspected angle closure glaucoma	27 (0.3)	28 (0.3)
Secondary ocular hypertension /suspected open angle glaucoma	2 (0.0)	4 (0.1)
Subtotal suspected glaucoma	540 (6.3)	542 (6.3)
Ocular hypertension	641 (7.4)	670 (7.8)
Primary angle closure	27 (0.3)	32 (0.4)
Narrow angles	36 (0.4)	34 (0.4)
Not recorded	23 (0.3)	26 (0.3)
Total	8623 (100)	8623 (100)

**Table 2 tbl2:** Diagnosis of glaucoma in 8623 men and women aged 48-92 in EPIC-Norfolk cohort. Figures are numbers (percentage) of participants

Diagnosis^*^	No (%) of participants
Normal	6713 (77.9)
Glaucoma	363 (4.2)
Suspected glaucoma	607 (7.0)
Ocular hypertension	863 (10.0)
Narrow angle spectrum	54 (0.6)
Unrecorded	23 (0.3)
Total	8623 (100)

*More serious diagnosis of either eye used, from (most serious to least serious): glaucoma, suspected glaucoma, ocular hypertension, narrow angle spectrum (primary angle closure, primary angle closure suspect), normal, diagnosis not recorded.

**Table 3 tbl3:** Type of glaucoma in 363 men and women aged 48-92 with glaucoma in EPIC-Norfolk cohort. Figures are numbers (percentage) of participants

Diagnosis	No (%) of participants
Primary open angle glaucoma	314 (86.5)
High tension glaucoma	157 (43.3)
Normal tension glaucoma	157 (43.3)
Primary angle closure glaucoma	29 (8.0)
Secondary glaucoma	20 (5.5)
Total (all glaucoma)	363 (100)

Among the cases of glaucoma, 242 (66.6%) were previously known, and 66.3% cases of primary open angle glaucoma were previously known. The prevalence of glaucoma in the study population increased with age and was higher in men than in women (table 4).

**Table 4 tbl4:** Glaucoma by age and sex in 363 men and women aged 48-92 with glaucoma in EPIC-Norfolk cohort. Figures are numbers (percentage of age group)

Age (years)	All cause glaucoma			Primary open angle glaucoma	
	Men	Women		Men	Women
<55	1 (0.8)	1 (0.5)		1 (0.8)	1 (0.5)
55-60	4 (1.5)	5 (1.0)		4 (1.5)	5 (1.0)
60-65	20 (2.3)	19 (1.5)		16 (1.8)	15 (1.2)
65-70	34 (4.3)	22 (2.2)		27 (3.4)	21 (2.1)
70-75	50 (6.6)	42 (5.0)		44 (5.8)	31 (3.7)
75-80	43 (7.2)	30 (4.9)		39 (6.6)	26 (4.3)
≥80	48 (11.2)	44 (10.8)		44 (10.5)	41 (10.1)
Total	200 (5.2)	163 (3.4)		175 (4.5)	140 (3.0)

IOP was measured in 8401 participants (7958 with ORA, 443 with AT555 NCT), 243 of whom used ocular hypotensive eye drops in either eye. Figure 3 shows the distribution of mean IOP of both eyes, which followed an approximately Gaussian distribution, with a right skew and an exaggerated peak. The cohort mean IOP was 16.3 mm Hg (95% confidence interval 16.2 mm Hg to 16.3 mm Hg; SD 3.6 mm Hg). Table 5 shows the distribution of IOP by age and sex. The mean IOP for glaucomatous eyes was 16.7 mm Hg (17.1 mm Hg to 18.1 mm Hg; range 4.0-45.6 mm Hg), and the percentage of eyes with glaucoma increased with IOP (fig 4). Of the 107 patients with a new diagnosis of primary open angle glaucoma, 76% (81) had mean IOP below 21 mm Hg.

**Figure f3:**
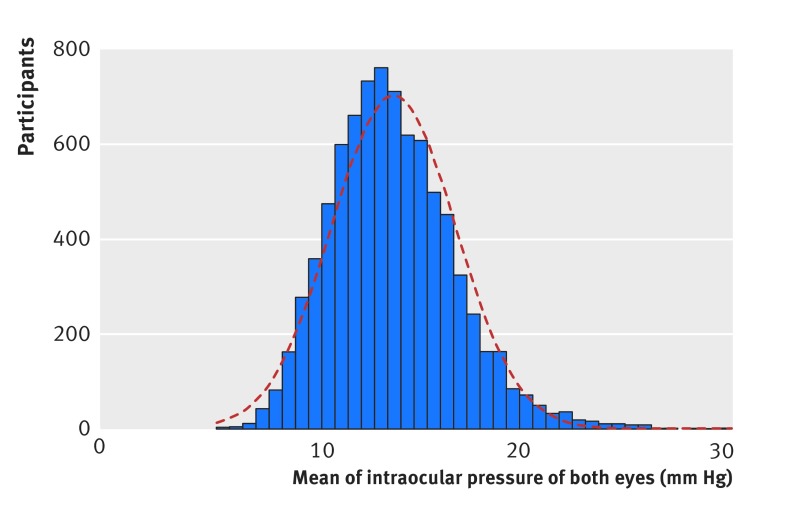
**Fig 3** Distribution of IOP in EPIC-Norfolk population (n=8401). Distribution approximates Gaussian distribution but has exaggerated central peak and modest right skew

**Table 5 tbl5:** Distribution of mean intraocular pressure (IOP)^*^ by age and sex in 8623 men and women aged 48-92 in EPIC-Norfolk cohort

Age group (years)	Men			Women	
	No of patients	IOP mm Hg (95% CI )		No of patients	IOP mm Hg (95% CI)
<55	128	15.9 (15.4 to 16.5)		185	15.7 (15.2 to 16.2)
55-<60	262	15.8 (15.4 to 16.3)		473	15.9 (15.6 to 16.2)
60-<65	857	16.4 (16.2 to 16.7)		1240	16.5 (16.3 to 16.6)
65-<70	790	16.2 (15.9 to 16.4)		969	16.7 (16.5 to 17.0)
70-<75	746	16.3 (16.0 to 16.5)		808	16.3 (16.1 to 16.6)
75-<80	570	16.0 (15.7 to 16.4)		591	16.2 (15.9 to 16.4)
≥80	402	16.0 (15.6 to 16.4)		380	15.8 (15.5 to 16.2)
Total	3755	16.2 (16.1 to 16.3)		4646	16.3 (16.2 to 16.4)

*Mean IOP of both eyes.

**Figure f4:**
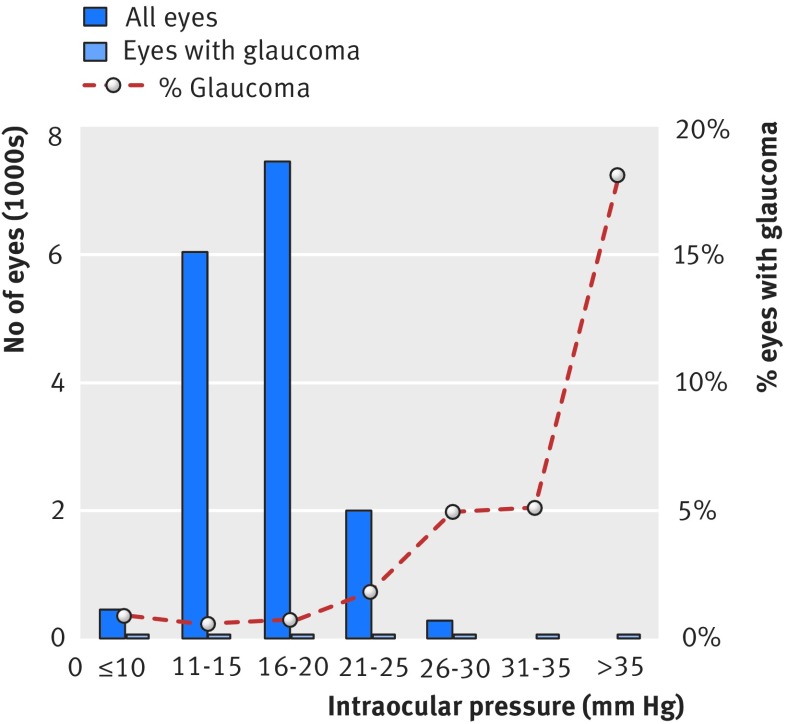
**Fig 4** Intraocular pressure for all eyes and eyes with glaucoma in EPIC-Norfolk cohort

Table 6 and figure 5 show the sensitivity and specificity of glaucoma detection at different IOP thresholds. Overall, sensitivity was poor at all levels shown, regardless of the additional refining parameters of age and sex, and there was no one single level that afforded both high sensitivity and specificity.

**Table 6 tbl6:** Sensitivity and specificity for detection of all cause glaucoma at different thresholds of intraocular pressure (IOP)

IOP mm Hg	Sensitivity (%)								Specificity (%)						
	Overall	Age				Men	Women		Overall	Age				Men	Women
		<65	≤65	<70	≥70					<65	≤65	<70	≥70		
>19	45.0	36.7	46.3	45.6	44.7	49.2	39.7		73.2	74.1	72.6	72.8	73.6	73.7	72.7
>20	36.3	26.5	37.9	34.0	37.3	42.4	28.9		81.0	82.0	80.3	80.9	81.0	80.5	81.3
>21	30.0	24.5	30.9	28.2	30.7	35.1	23.7		86.9	87.7	86.4	86.8	87.0	85.8	87.7
>22	25.4	22.5	25.8	23.3	26.2	30.4	19.2		91.2	91.9	90.7	91.1	91.3	90.3	91.9
>23	20.5	18.4	20.8	20.4	20.5	24.6	15.4		94.0	94.5	93.8	93.8	94.5	93.2	94.7
>24	16.7	18.4	16.4	16.5	16.8	20.9	11.5		96.0	96.2	95.9	95.7	96.4	95.4	96.5
>25	12.1	12.2	12.1	10.7	12.7	16.2	7.1		97.1	97.0	97.2	96.9	97.5	96.6	97.6
>26	7.8	8.2	7.7	6.8	8.2	11.0	3.9		98.0	97.8	98.1	97.8	98.3	97.5	98.4

**Figure f5:**
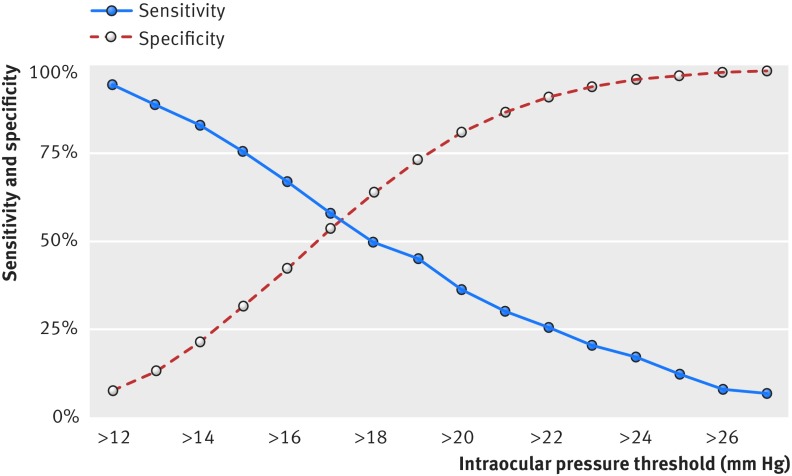
**Fig 5** Sensitivity and specificity for detection of all cause glaucoma in EPIC-Norfolk cohort

## Discussion

In this large population based study, we found that intraocular pressure was not a sensitive or specific indicator of glaucoma. This is the most current large scale population study reporting glaucoma epidemiology in the UK. We found many participants with suspected glaucoma or ocular hypertension, confirming a large potential referral burden to the NHS. IOP has also been shown to be a poor case finding test for glaucoma. 

### Principal findings and comparison with other studies

Data on prevalence of glaucoma have been reported from populations in the US,^16^ ^17^ Australia,^18^ ^19^ Europe,^20^ ^21^ ^22^ and South East Asia.^23^ ^24^ ^25^ ^26^ Recent data from the UK, however, is lacking, with the last published cross sectional population surveys being one from rural west of Ireland in 1993^27^ and another from north London in 1998.^28^

There were differences between the participants from EPIC-Norfolk and the local population of Norfolk as the study participants were not sampled systematically but recruited by inviting all adults aged >40 from GP practices. Apart from differences in age and sex composition, EPIC-Norfolk participants were less likely to live in deprived areas and were potentially healthier because of the volunteer nature of the study. The people with glaucoma identified in the cohort might therefore not be fully representative of the local or national population and are probably an underestimation of the true numbers. Nevertheless, results in this study corroborated many established trends in glaucoma epidemiology. The predominant type in our cohort was primary open angle glaucoma, a consistent finding among European populations.^5^ ^29^ The prevalence increased with age, which is its strongest known risk factor.^30^ The prevalence of all cause glaucoma in those aged 48-92 was 4.2% (95% confidence interval 3.8% to 4.6%) and 3.7% (3.3% to 4.0%) for primary open angle glaucoma. This echoed findings from a meta-analysis in 2014, in which the prevalence of glaucoma (primary open angle glaucoma and primary angle closure glaucoma) for Europeans aged 40-80 was 2.93% (1.85% to 4.40%) and the prevalence of primary open angle glaucoma was 2.51% (1.54% to 3.89%).^5^ In another meta-analysis, published in 2006, the pooled prevalence of primary open angle glaucoma in white people was 2.1% (1.6% to 2.7%).^31^

In our cohort, two thirds of those with primary open angle glaucoma had previously received the diagnosis. This is the highest reported figure from a major community based study. Previous reported figures include 49% in the Blue Mountains Eye Study,^18^ 50% in Melbourne’s Visual Impairment Study,^19^ 50% in the Thessaloniki Eye Study,^22^ 47% in the Rotterdam Eye Study,^20^ and 50% among white people in the Baltimore Eye Survey.^32^ Glaucoma is a largely asymptomatic disease, with insidious onset. In most industrialised countries, it is detected by opportunistic case finding and relies on people being examined by an eye care professional. In the UK, this would usually be a community optometrist. People with suspected glaucoma are then referred to ophthalmologists for definitive diagnosis and management. The higher rate of previously known glaucoma cases in EPIC-Norfolk than in other studies could reflect either better access to healthcare among the study participants because of recruitment bias or generally more effective provision of healthcare in the UK, with universal access and free eye tests for those aged over 60 in the NHS.

A striking finding in the study was the large number of people with suspected glaucoma (7%) and ocular hypertension (10%). Collectively they represent a large number of potential referrals to the hospital eye services, many of whom remain under observation for up to five years.^33^ This is reflected by the existing burden in hospital eye services, whereby ocular hypertension accounts for 30-45% of the referrals it receives.^34^ ^35^ Coupled with the fact that glaucoma is a chronic disease that needs regular and long term follow-up, it is no wonder that glaucoma and suspected glaucoma account for the sixth largest share of NHS outpatient attendances.^4^

While raised IOP is the strongest risk factor after age for primary open angle glaucoma,^30^ our data reiterate that no single IOP level provides sufficiently high sensitivity and specificity for detection of glaucoma, as shown in figure 3, mirroring results from the Baltimore Eye Survey.^16^ This reinforces the principle that IOP alone without optic disc examination or a visual field test is not an effective screening tool for glaucoma.

### Limitations of study 

There were several sources of under-reporting of a diagnosis of glaucoma in this study. Only 18% of participants underwent visual field testing. A meta-analysis showed that lack of routine field testing in a population study was a study design factor that led to underdiagnosis.^36^ In our study, however, both disc and field abnormalities were prerequisites of diagnosis, supporting well established diagnostic principles used in most population cross sectional studies.^17^ ^20^ ^23^ ^32^ ^37^ ^38^ We used a multimodal optic disc examination to uncover glaucomatous damage and determine who was referred for a definitive exam. We therefore expect that few cases of glaucoma would have been missed. The number of cases of narrow angle spectrum is also likely to be underestimated, as gonioscopy or anterior chamber depth assessment on slit lamp were not part of the screening test, although those with primary open angle glaucoma should not have been missed because of that as all glaucoma suspects underwent a full examination.

### Conclusion

In conclusion, this study confirms the high prevalence of glaucoma and suspected glaucoma in the UK. We have reported the IOP distribution among the population and among those with glaucoma, confirming its poor case finding performance. These findings will be useful in the planning of ophthalmic services in the UK and help to revaluate the use of IOP in making referrals from the community to the hospital eye services.

**What is already known on this topic**

Glaucoma is the leading cause of irreversible blindness in the world and the second most common cause of registered blindness in England and Wales
The management of glaucoma, suspected glaucoma, and ocular hypertension accounts for a considerable amount of NHS outpatient resources
While the prevalence of glaucoma has been reported in many population studies worldwide, there are no recent data for the UK

**What this study adds**

This study provides the most current data on prevalence and type of glaucoma in a British community and identified a large number of people with ocular hypertension and suspected glaucoma
The large number of people with confirmed glaucoma and intraocular pressure under the threshold for ocular hypertension (21 mm Hg) reinforces the weakness of reliance on this for detection of glaucoma

## Notes

We thank Haogang Zhu for his help in extracting visual field data, Pak Sang Lee for his technical assistance with the EPIC-Norfolk Eye Study, and Ananth Viswanathan FRCOphth for providing one of the visual field analysers used in this study.

Contributors: MPYC analysed and interpreted the data and drafted the manuscript. PJF and DCB contributed to the conception and design of the study and to data collection. APK and JLYY contributed to data collection and interpretation. DGH contributed to the conception and design of the study and to data interpretation. JMB contributed to data interpretation. RL contributed to the design of the study and to data management. SH contributed to the design of the study. ND contributed to the design of the study and to data acquisition. KTK contributed to the conception and design of the study and to data interpretation. All authors read and critically revised the manuscript and approved the final manuscript. MPYC and PJF are guarantors.

Ethical approval: The work was approval by the East Norfolk and Waverney NHS research governance committee (2005EC07L) and the Norfolk research ethics committee (05/Q0101/191).

Data sharing: Requests for data sharing/access should be submitted to the EPIC Management Committee via Mrs Shabina Hayat (sah63@medschl.cam.ac.uk). Applications will be judged on a case by case basis, determined by the scientific merit, in line with MRC guidelines (https://www.mrc.ac.uk/publications/browse/mrc-policy-and-guidance-on-sharing-of-research-data-from-population-and-patient-studies/).

Transparency: The lead author (the manuscript’s guarantor) affirms that the manuscript is an honest, accurate, and transparent account of the study being reported; that no important aspects of the study have been omitted; and that any discrepancies from the study as planned (and, if relevant, registered) have been explained.
